# Long non-coding RNA C5orf66-AS1 promotes cell proliferation in cervical cancer by targeting miR-637/RING1 axis

**DOI:** 10.1038/s41419-018-1228-z

**Published:** 2018-12-05

**Authors:** Xiaohui Rui, Yun Xu, Xiping Jiang, Wenfeng Ye, Yaqing Huang, Jingting Jiang

**Affiliations:** 1 Department of Gynecology, The Third Affiliated Hospital of Soochow University, Changzhou, 213003 People’s Republic of China; 2 Jiangsu Engineering Research Center for Tumor Immunotherapy, Changzhou, 213003 People’s Republic of China; 3 Institute of Cell Therapy, Soochow University, Changzhou, 213003 People’s Republic of China; 4 Department of Tumor Biological Treatment, The Third Affiliated Hospital of Soochow University, 185 Juqian Street, Changzhou, 213003 Jiangsu China

## Abstract

Long non-coding RNA (lncRNA) plays an important role in the development of human malignant tumours. Recently, an increasing number of lncRNAs have been identified and investigated in a variety of tumours. However, the expression pattern and biological function of lncRNAs in cervical cancer still remain largely unexplored. Differentially expressed lncRNAs in cervical cancer and para-carcinoma tissues were identified by screening using The Cancer Genome Atlas (TCGA), and candidate lncRNAs were verified by quantitative real-time PCR. We found that lncRNAC5orf66-AS1 was significantly upregulated in cervical cancer tissues and cells. Over-expression of C5orf66-AS1 promoted the proliferation of cervical cancer cells, while downregulation of C5orf66-AS1 promoted the apoptosis of cervical cancer cells. C5orf66-AS1 was identified as the sponge of miR-637 by RNA immunoprecipitation (RIP) and luciferase reporter assays. Exogenous miR-637 and RING1 interventions could reverse the proliferation ability mediated by C5orf66-AS1 in cervical cancer cells. In vivo experiments also confirmed that downregulation of C5orf66-AS1 inhibited the tumour growth. LncRNA C5orf66-AS1, as a competitive endogenous RNA (ceRNA), regulated the effect of RING1 on the proliferation, apoptosis and cell cycle of cervical cancer cells through adsorbing miR-637. Taken together, our findings provided a new theoretical and experimental basis for investigating the pathogenesis and exploring effective therapeutic targets for cervical cancer.

## Introduction

As one of the most common gynaecological malignant tumours, cervical cancer has become an important public health issue. The incidence rate of cervical cancer has been reported to rank the 2nd in the world among female malignant tumours, and its mortality rate ranks the 1st among female malignant tumours of the reproductive system, rendering it a disease that seriously threatens female health^1^. According to the statistics, there were approximately 530,000 new cases of cervical cancer in the world in 2008, 85% of which occurred in developing countries and approximately one-third occurred in China^2^. At present, surgery, chemotherapy and radiotherapy are the predominant therapeutic schemes for cervical cancer, but most cervical cancer cells are resistant to chemotherapeutic drugs, resulting in a poor therapeutic effect^3^. There is a lack of an effective therapeutic method for advanced and recurrent cervical cancer with poor prognosis. Therefore, it is urgently necessary to investigate new treatments of cervical cancer. However, only few studies have investigated the exact mechanism of cervical cancer, greatly limiting the development of molecular-targeted drug therapies. Therefore, further studies on the molecular mechanism of cervical cancer and the development of new molecular targets have become research hotspots.

With the development of the human genome sequencing technique, it has been reported that the proportion of protein-coding genes in the total DNA sequence of the human genome is less than 2%. More than 98% of the sequences are RNAs without the protein-coding function^4^, known as non-coding RNA. Non-coding RNAs are divided into long and short non-coding RNAs based on sequence lengths^5^. Long non-coding RNA (lncRNA) is a type of non-coding RNA with more than 200 nucleotides, and it possesses similar structural features to mRNA. Most of the lncRNAs are produced via RNA polymerase II transcription^6^. Although lncRNA does not encode a protein, it can affect the expression levels of a variety of genes at the transcriptional and post-transcriptional levels^7^. According to recent studies, the expression of lncRNA is closely related to various tumours, such as colon cancer^8^, breast cancer^9^ and liver cancer^10^. However, the mechanism of lncRNA in cervical cancer still remains largely unexplored.

In the present study, differentially expressed lncRNAs were identified in three pairs of cervical cancer tissues and corresponding para-carcinoma tissues using The Cancer Genome Atlas (TCGA) database. Five pairs of lncRNAs that were upregulated and downregulated were verified via quantitative real-time reverse transcription PCR (qRT-PCR). Finally, lncRNA C5orf66-AS1 was selected as the object of our current study. Up- and downregulation of lncRNA C5orf66-AS1 in vitro and in vivo affected the biological behaviour of cervical cancer. Therefore, it could be used to explore the target genes of lncRNA C5orf66-AS1 in the proliferation of cervical cancer. Taken together, our findings provided a new theoretical basis for the effective prevention and treatment of cervical cancer.

## Results

### C5orf66-AS1 is highly expressed in cervical cancer

**Fig. 1:**
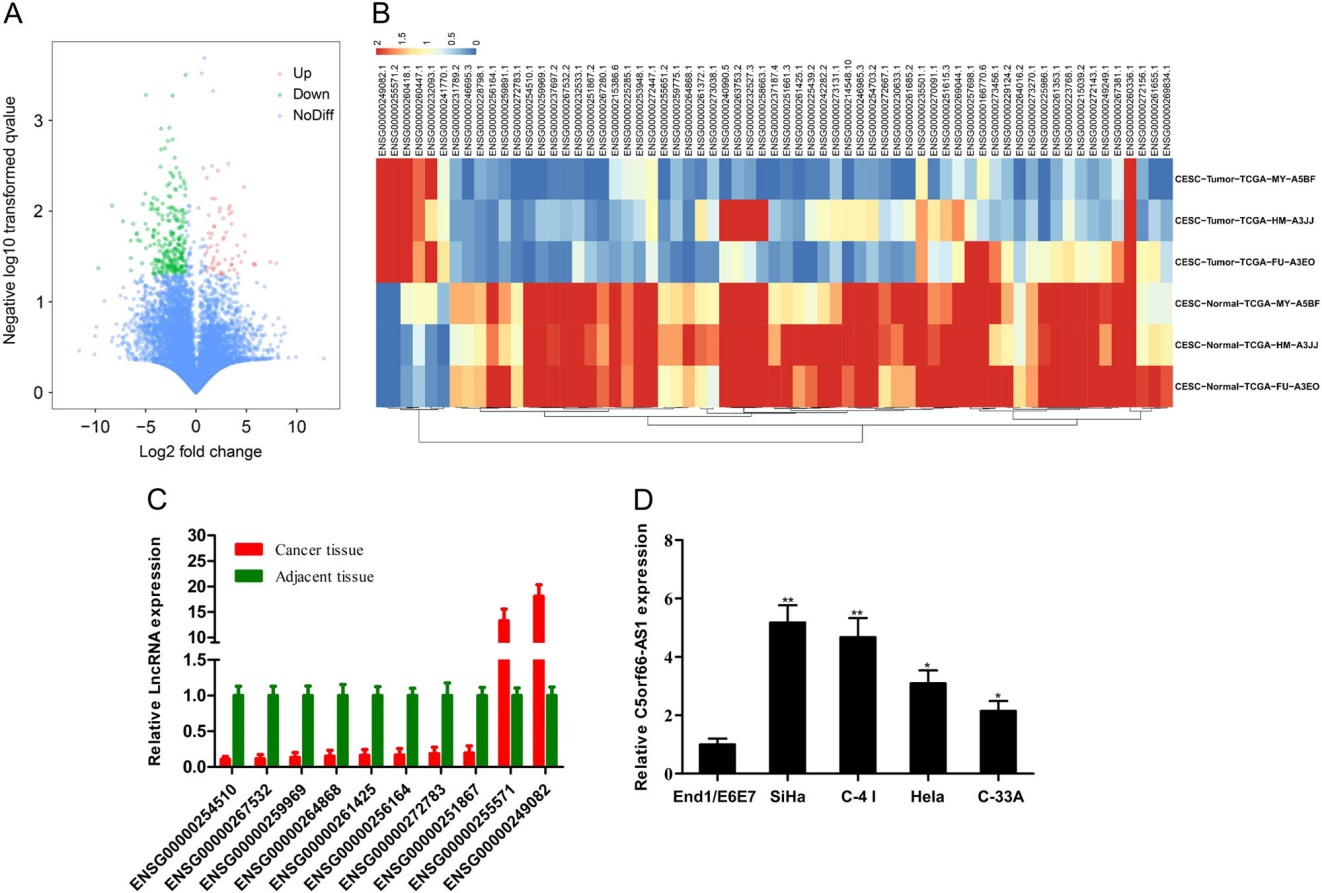
Screening of differentially expressed lncRNAs in cervical cancer. **a** Volcano plots were constructed based on TCGA (fold change >2.0 and *P* < 0.05). The red points represent differentially upregulated genes, and green points represent downregulated genes. **b** Analysis of differentially expressed genes in cervical cancer based on TCGA (fold change >2.0, *P* < 0.05 in *t*-test, an average FPKM greater than 1 in carcinoma or para-carcinoma tissues). **c** Validation of lncRNAs in 20 pairs of cervical cancer tissues and para-carcinoma tissues via qRT-PCR. ENSG00000249082 (C5orf66-AS1) exhibited the most significant difference in cervical cancer tissues. **d** The expression of lncRNA C5orf66-AS1 in the cervical cancer cell lines SiHa, C-4 I, C-33A and HeLa were detected via qRT-PCR using the normal cervical epithelial cells End1/E6E7 as a control. ^*^P < 0.05, ^**^P < 0.01, ^***^P < 0.001

The lncRNA expression profile and genomic information of 13 types of tumours were comprehensively analyzed at the MD Anderson Cancer Research Center using TCGA. The sequencing data of lncRNAs in three pairs of cervical cancer and para-carcinoma tissues were downloaded and analyzed. The expressions of lncRNAs in cervical cancer were found to be primarily depleted or downregulated (259 downregulated lncRNAs and 77 upregulated lncRNAs) based on a threshold of a > 2.0 fold change in expression between cervical cancer and para-carcinoma tissues (*P* < 0.05 in *t*-test) (Fig. 1a). However, most lncRNAs were poorly expressed in cancer and para-carcinoma tissues. Therefore, only lncRNAs with an average FPKM > 1 in cancer or para-carcinoma tissues were selected for the subsequent study. A total of 65 lncRNAs (59 downregulated lncRNAs and six upregulated lncRNAs) met the requirements of the study (Fig. 1b). A total of 10 differentially expressed lncRNAs (ENSG00000254510, ENSG00000267532, ENSG00000259969, ENSG00000264868, ENSG00000261425, ENSG00000256164, ENSG00000272783, ENSG00000251867, ENSG00000255571 and ENSG00000249082) were selected for qRT-PCR in 20 pairs of cervical cancer tissues and para-carcinoma tissues. The results revealed that ENSG00000249082 (C5orf66-AS1) exhibited the most significant difference in cervical cancer tissues (Fig. 1c). In addition, the expression of lncRNA C5orf66-AS1 in the cervical cancer cell lines SiHa, C-4 I, C-33A and HeLa were detected via qRT-PCR with the normal cervical epithelial cells End1/E6E7 as a control. The expression of C5orf66-AS1 in cervical cancer cell lines was higher than that in normal cell line, and its highest expression was detected in SiHa cells (HPV-16-infected cervical cancer cell line) and C-4 I cells (HPV-18-infected cervical cancer cell line) (Fig. 1d).

### LncRNA C5orf66-AS1 promotes the proliferation of cervical cancer cells

**Fig. 2:**
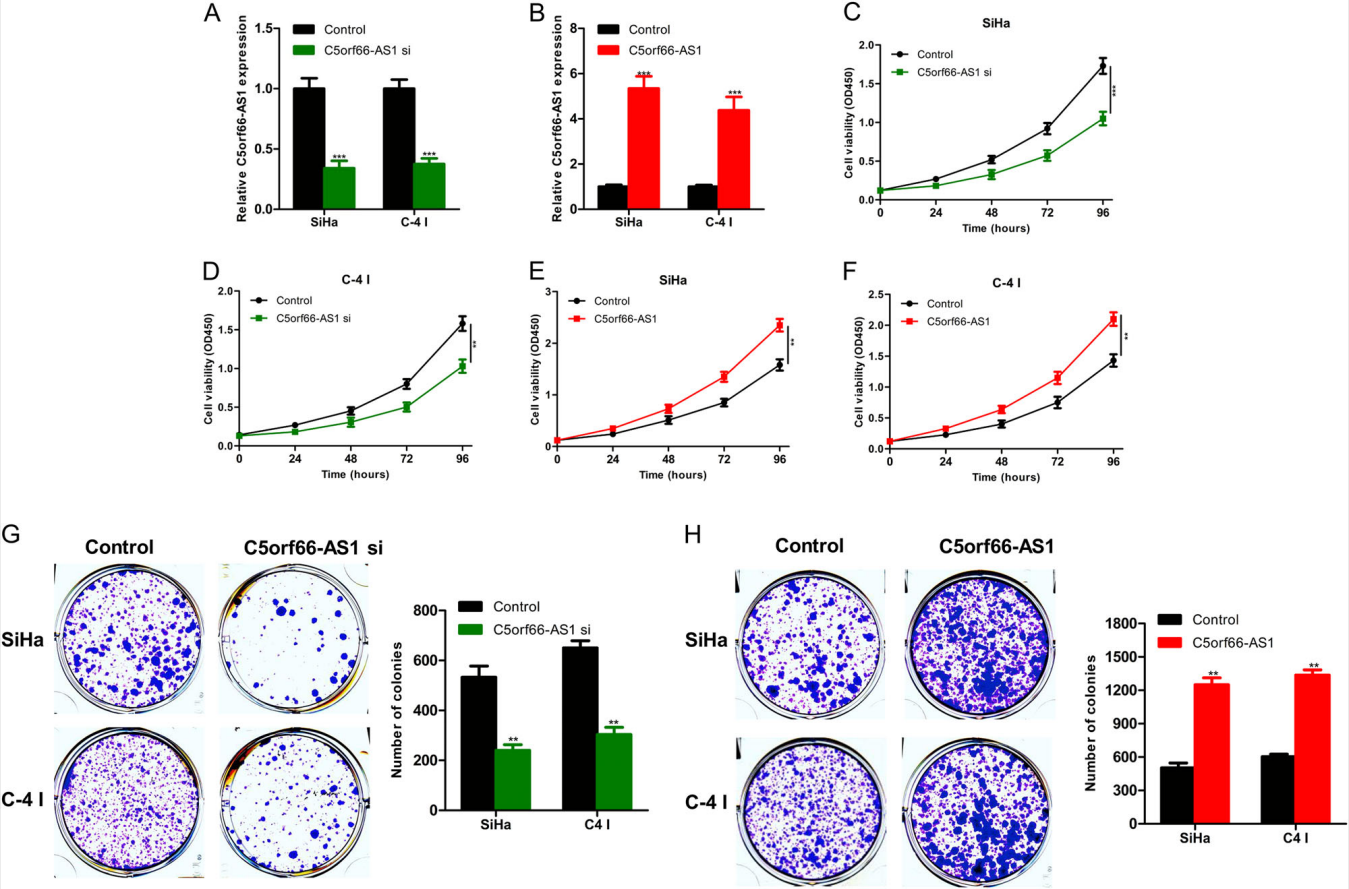
LncRNA C5orf66-AS1 can promote the proliferation of cervical cancer cells. **a** After downregulation of C5orf66-AS1 in SiHa and C-4 I cell lines, interference efficiency was verified via qRT-PCR. **b** After upregulation of C5orf66-AS1 in SiHa and C-4 I cell lines, interference efficiency was verified via qRT-PCR. After downregulation of C5orf66-AS1, CCK8 assay revealed that the proliferation of **c** SiHa and **d** C-4 I cells was significantly decreased. After upregulation of C5orf66-AS1, CCK8 assay revealed that the proliferation of **e** SiHa and **f** C-4 I cells was significantly enhanced. **g** After downregulation of C5orf66-AS1, colony-formation assay revealed that the proliferation of SiHa and C-4 I cells was significantly decreased. **h** After upregulation of C5orf66-AS1, colony-formation assay revealed that the proliferation of SiHa and C-4 I cells was significantly enhanced. ^*^P < 0.05, ^**^P < 0.01, ^***^P < 0.001

**Fig. 3:**
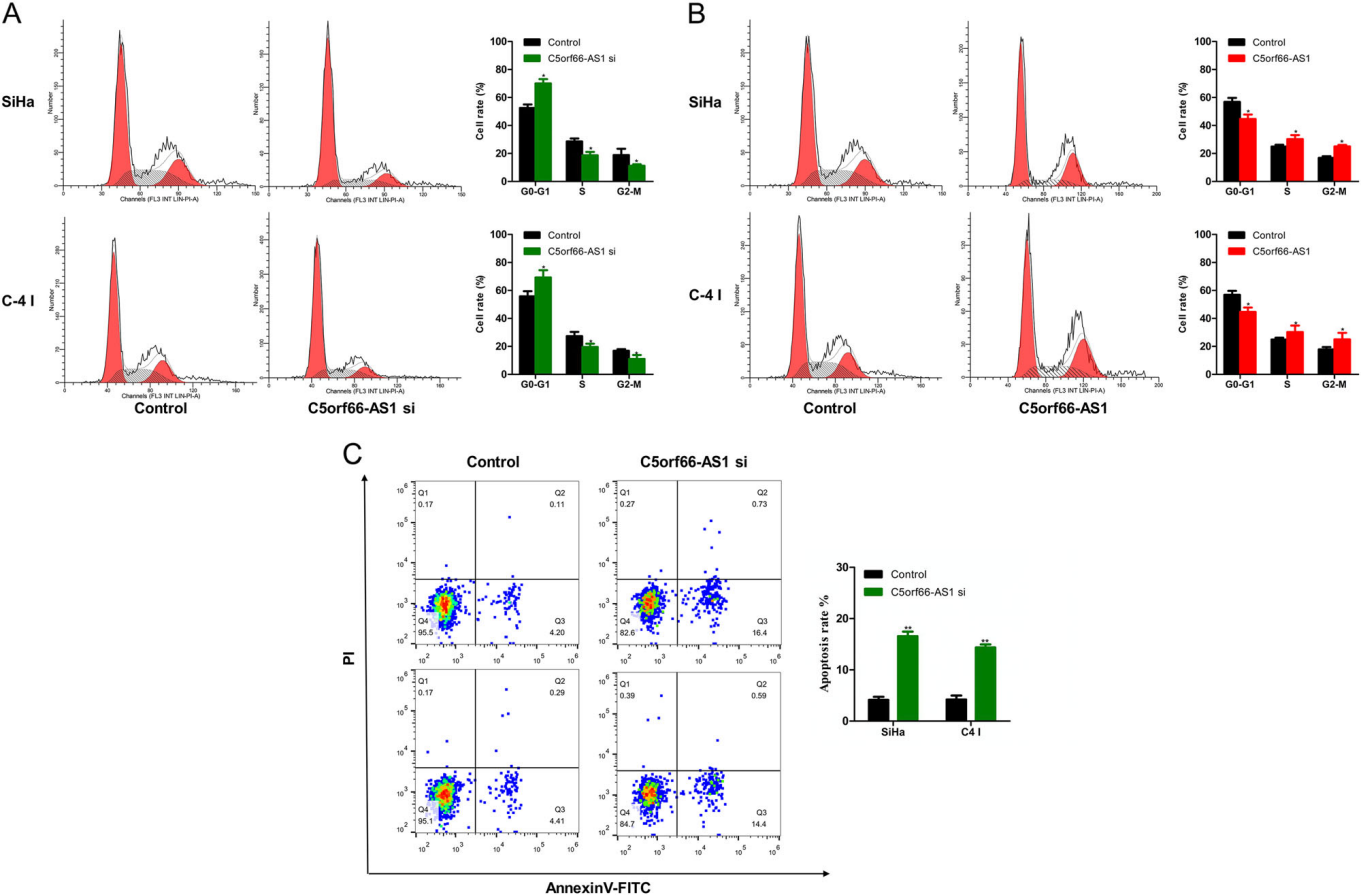
LncRNA C5orf66-AS1 can regulate the cell cycle and apoptosis of cervical cancer cells. **a** The cell cycle was analyzed via flow cytometry. The number of cells in the G1/G0 phase was increased with downregulation of C5orf66-AS1, while that in the G2/S phase was declined. **b** The number of cells in the G1/G0 phase was declined obviously with upregulation of C5orf66-AS1, while that in the G2/S phase was significantly increased. **c** The apoptotic rates of cells were analyzed via flow cytometry. Downregulation of C5orf66-AS1 promoted apoptosis of cervical cancer cells. ^*^P < 0.05, ^**^P < 0.01, ^***^P < 0.001

To explore the influence of changes in C5orf66-AS1 expression on the proliferation of cervical cancer cells, C5orf66-AS1 was down- or upregulated in cervical cancer SiHa and C-4 I cell lines via transfection with siRNA or the pcDNA3.1-C5orf66-AS1 over-expression plasmid. Transfection with the C5orf66-AS1 over-expression plasmid or siRNA significantly up- or downregulated the expression of C5orf66-AS1, respectively (Figs. 2a, b). Cell proliferation was detected via CCK-8 assay, and the data showed that after the suppression of C5orf66-AS1 in SiHa and C-4 I cells, cell proliferation was significantly decreased (Figs. 2c, d). However, cell proliferation was significantly increased after the upregulation of C5orf66-AS1 in SiHa and C-4 I cells (Figs. 2e, f). The same results were also obtained via the colony-formation assay (Figs. 2g, h). Moreover, the cell-cycle assay showed that the number of cells in the G1/G0 phase was increased with suppression of C5orf66-AS1, while that in the G2/S phase was reduced (Fig. 3a). The number of cells in the G1/G0 phase was decreased obviously with upregulation of C5orf66-AS1, while that in the G2/S phase was significantly increased (Fig. 3b). These results indicated that C5orf66-AS1 could regulate the cell cycle. In addition, downregulated C5orf66-AS1 expression promoted the apoptosis of cervical cancer cells (Fig. 3c). Due to the low rate of cancer cell apoptosis, upregulated C5orf66-AS1 did not significantly affect the apoptosis of cervical cancer cells.

### LncRNA C5orf66-AS1 plays a role as a competitive endogenous RNA (ceRNA) in regulating RING1 expression by binding to miR-637

**Fig. 4:**
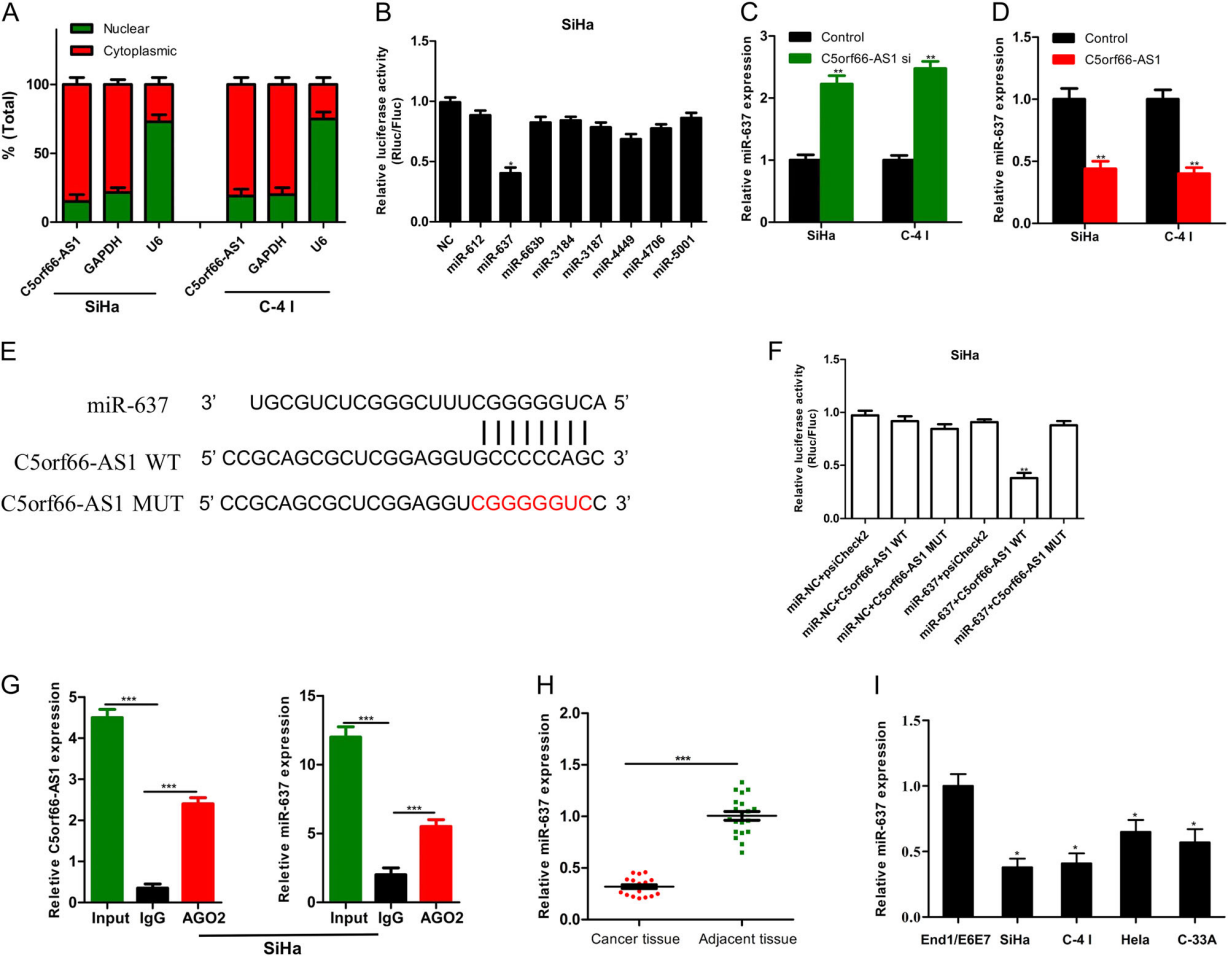
Regulation of screening and verification of miRNAs by C5orf66-AS1. **a** qRT-PCR analysis of C5orf66-AS1 expression at the nuclear and cytoplasmic levels in SiHa and C-4 I cells. U6 was used as a nuclear marker, and GAPDH was used as a cytosolic marker. **b** Relative luciferase activity from SiHa cells co-transfected with C5orf66-AS1 reporter plasmid and candidate miRNAs. **c** After downregulation of C5orf66-AS1 in SiHa and C-4 I cell lines, the miR-637 expression was significantly elevated. **d** After upregulation of C5orf66-AS1 in SiHa and C-4 I cell lines, the miR-637 expression was significantly reduced. **e** MiR-637 and C5orf66-AS1 binding sequences and C5orf66-AS1 mutation sequences. **f** Luciferase reporter assay showed that over-expression of miR-637 significantly reduced the luciferase activity in cells that were transfected with the wild-type C5orf66-AS1 vector without reducing the luciferase activity in cells that were transfected with the mutant-type vector or empty vector. **g** RIP experiments were performed in SiHa cells, and the co-precipitated miR-637 was subjected to qRT-PCR for lncRNA C5orf66-AS1. **h** Expression of miR-637 was significantly decreased in cervical cancer tissues compared with adjacent normal tissues. **i** The expression of miR-637 in the cervical cancer cell lines SiHa, C-4 I, C-33A and HeLa were detected via qRT-PCR using the normal cervical epithelial cells End1/E6E7 as a control. ^*^P < 0.05, ^**^P < 0.01, ^***^P < 0.001

CeRNA is an important regulatory mechanism of lncRNA. CeRNA theory argues that lncRNA, which is highly expressed in the cytoplasm, can competitively bind to miRNA and regulate its downstream target genes, thereby inhibiting the biological function of miRNAs and participating in tumour development. Through the nuclear-cytoplasmic fractionation assay, C5orf66-AS1 was mainly located in the cytoplasm (Fig. 4a). Therefore, C5orf66-AS1 was speculated to regulate the function of cervical cancer cells through the ceRNA mechanism. The RegRNA2.0 database (http://regrna2.mbc.nctu.edu.tw/) revealed that C5orf66-AS1 could interact with miR-621, miR-637, miR-663b, miR-3184, miR-3187, miR-4449, miR-4706 and miR-5001 via complementary base pairing. Therefore, C5orf66-AS1 was hypothesized to serve as a sponge for these miRNAs. The binding ability of C5orf66-AS1 to candidate miRNAs was further verified in SiHa cells using an immunofluorescence reporter assay, and miR-637 was used as a candidate miRNA (Fig. 4b). Next, the expression of C5orf66-AS1 was upregulated or downregulated in cervical cancer cells SiHa and C-4 I, and the expression of miR-637 was detected by qRT-PCR. Downregulation of C5orf66-AS1 significantly promoted the expression of miR-637 compared with the control treatment (Fig. 4c). However, upregulation of C5orf66-AS1 significantly decreased the expression of miR-637 (Fig. 4d). We next constructed the fluorescent reporter enzyme plasmids C5orf66-AS1-WT and C5orf66-AS1-MUT with a miR-637 binding site (Fig. 4e). Upregulation of miR-637 significantly reduced the luciferase activity in SiHa cells co-transfected with C5orf66-AS1-WT, while upregulation of miR-637 had no effect on luciferase activity when the cells were co-transfected with C5orf66-AS1-MUT, suggesting that C5orf66-AS1 bound directly to miR-637 (Fig. 4f). The RIP assay was used to validate the potentially endogenous interaction between C5orf66-AS1 and miR-637. The results showed that C5orf66-AS and miR-637 were preferentially enriched in the anti-Ago2 group compared with the IgG control group (Fig. 4g). Moreover, the expression of miR-637 was examined in 20 cases of cervical cancer and para-carcinoma tissues via qRT-PCR, and the miR-637 expression was low in cervical cancer (Fig. 4h). The expression of miR-637 in cervical cancer cell lines was lower than that in normal cervical epithelial cell lines (Fig. 4i).

**Fig. 5:**
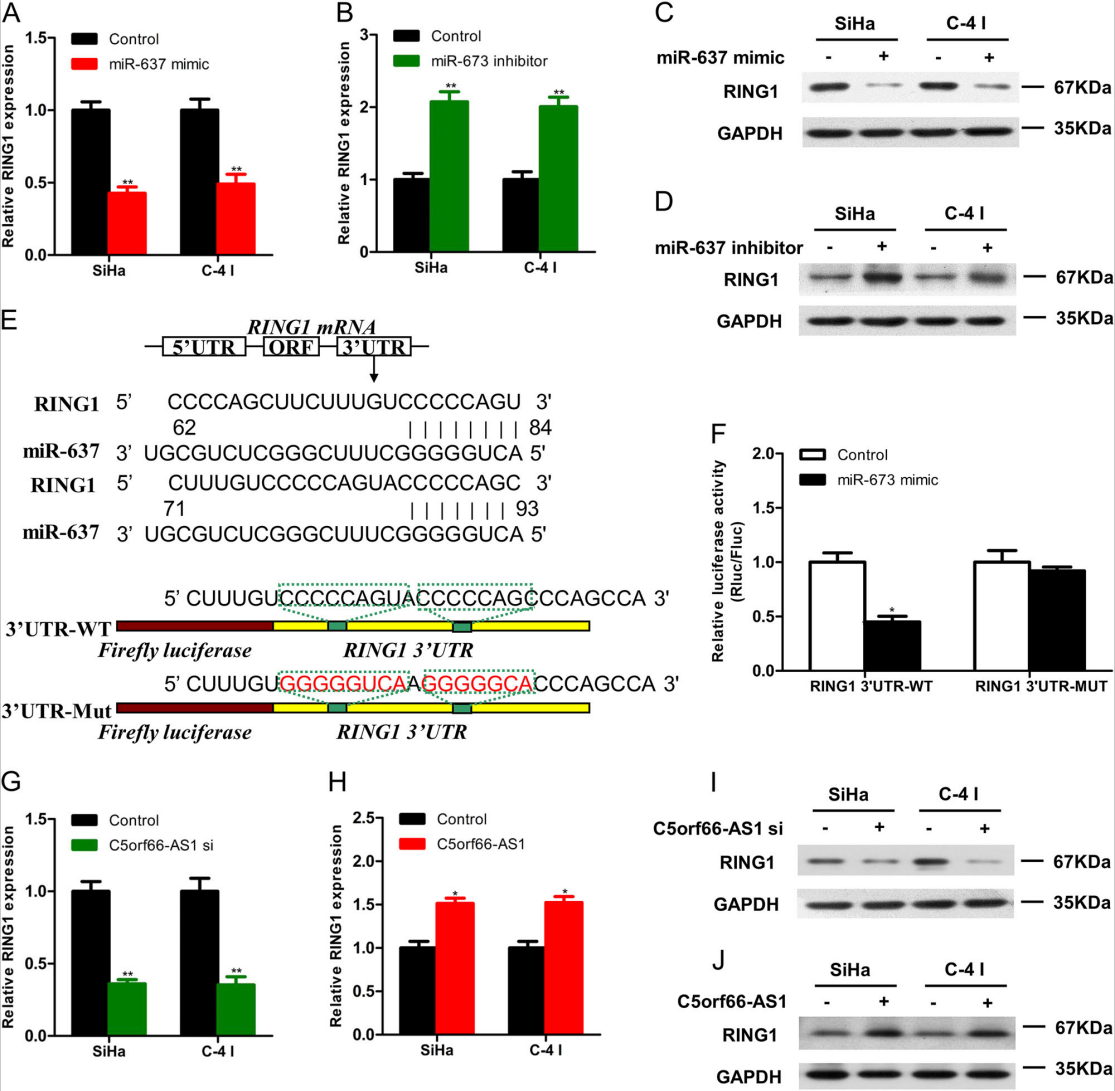
Screening and validation of miR-637 target genes. **a** After upregulation of miR-637 in SiHa and C-4 I cell lines, the expression of RING1 at the mRNA level was significantly decreased. **b** After downregulation of miR-637 in SiHa and C-4 I cell lines, the expression of RING1 at the mRNA level was significantly increased. **c** After upregulation of miR-637 in SiHa and C-4 I cell lines, the expression of RING1 at the protein level was significantly decreased. **d** After upregulation of miR-637 in SiHa and C-4 I cell lines, the expression of RING1 at the protein level was significantly increased. **e** MiR-637 and RING1 3′UTR binding sequences and RING1 mutation sequences. **f** Luciferase reporter assay showed that transfection with wild-type RING1 plasmid significantly reduced the fluorescence intensity, while the fluorescence intensity after transfection with mutant-type RING1 plasmid exhibited no significant change. **g** After downregulation of C5orf66-AS1 in SiHa and C-4 I cell lines, the expression of RING1 at the mRNA level was significantly decreased. **h** After upregulation of C5orf66-AS1 in SiHa and C-4 I cell lines, the expression of RING1 at the mRNA level was significantly increased. **i** After downregulation of C5orf66-AS1 in SiHa and C-4 I cell lines, the expression of RING1 at the protein level was significantly decreased. **j** After upregulation of C5orf66-AS1 in SiHa and C-4 I cell lines, the expression of RING1 at the protein level was significantly increased. ^*^P < 0.05, ^**^P < 0.01, ^***^P < 0.001

The target genes of miR-637 were predicted based on the TargetScan (http://www.targetscan.org/) and miRDB (http://mirdb.org/) databases, and SLC8A2, SPRED3, TSPAN11, MNT, PVRL1 and RING1 were used as candidate target genes. The expression of miR-637 in SiHa and C-4 I cells was increased or decreased, and only the expression of RING1 at the mRNA and protein levels was changed (Figs. 5a–d). Next, we constructed the fluorescent reporter enzyme plasmids RING1 3′UTR-WT and RING1 3′UTR-MUT with the miR-637 binding site (Fig. 5e). The results of the immunofluorescence reporter assay revealed that SiHa cells co-transfected with the RING1 3′UTR-MUT plasmid and miR-637 showed significantly less luciferase activity compared with the control group, and the upregulation of miR-637 exhibited did not affect the luciferase activity of cells that were co-transfected with the RING1 3′UTR-MUT plasmid, suggesting that RING1 could bind directly to miR-637 (Fig. 5f). In addition, after up- or downregulation of C5orf66-AS1 in SiHa and C-4 I cell lines, the expression of RING1 at the mRNA and protein levels was significantly increased or decreased, respectively (Fig. 5g–j). These above-mentioned results indicated that C5orf66-AS1, miR-637 and RING1 acted together in a ceRNA mechanism.

**Fig. 6:**
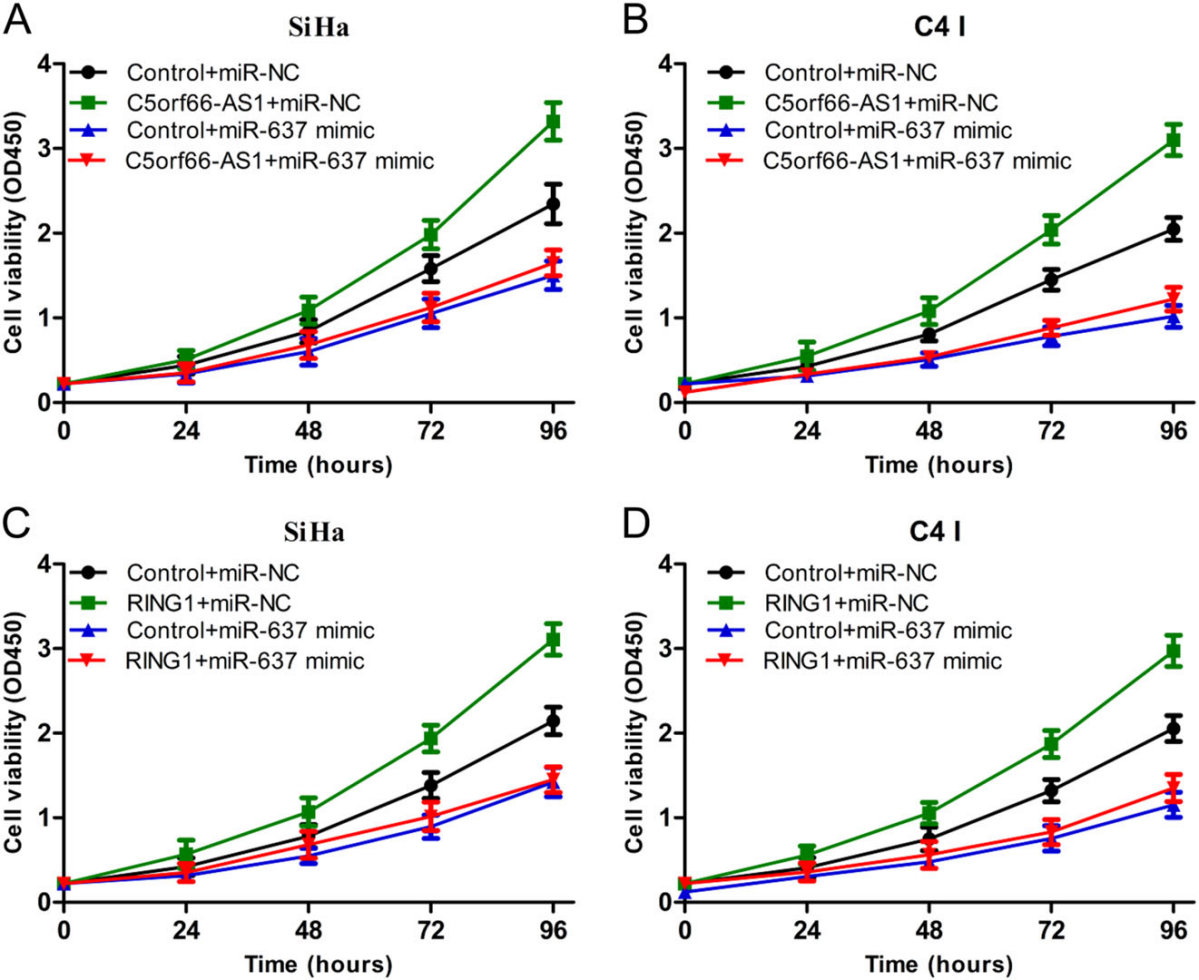
The regulation of proliferation of cervical cancer by C5orf66-AS1 depends on the miR-637/RING1 pathway. **a**, **b** Growth curves for SiHa and C-4 I cells after co-transfection with C5orf66-AS1, miR-637 mimic or control were determined via the CCK8 assay. **c**, **d** Growth curves for SiHa and C-4 I cells after co-transfection with RING1, miR-637 or control were determined via CCK8 assay. ^*^P < 0.05, ^**^P < 0.01, ^***^P < 0.001

To verify whether C5orf66-AS1 regulated the proliferation of cervical cancer cells depending on the miR-637/RING1 pathway, the following rescue experiments were carried out. First, SiHa and C-4 I cells were divided into four groups: (1) control + miR-NC, (2) C5orf66-AS1 + miR-NC, (3) control + miR-637 mimic, and (4) C5orf66-AS1 + miR-637 mimic. The over-expression of miR-637 alone significantly inhibited the proliferation of SiHa and C-4 I cells, and miR-637 partially reversed such alterations caused by C5orf66-AS1 via over-expression of C5orf66-AS1 or upregulation of miR-637 (Fig. 6a, b). Moreover, the SiHa or C-4 I cells were divided into four groups as follows: (1) control + miR-NC, (2) RING1 + miR-NC, (3) control + miR-637 mimic and (4) RING1 + miR-637 mimic. The over-expression of RING1 alone significantly enhanced the proliferation of SiHa and C-4 I cells, and RING1 completely reversed these changes caused by miR-637 via over-expression of RING1 or upregulation of miR-637 (Fig. 6c, d). In summary, C5orf66-AS1 was believed to adsorb miR-637 through a ceRNA mechanism and upregulate RING1, ultimately promoting the proliferation of cervical cancer cells.

### Downregulation of lncRNA C5orf66-AS1 inhibits tumour growth in vivo

**Fig. 7:**
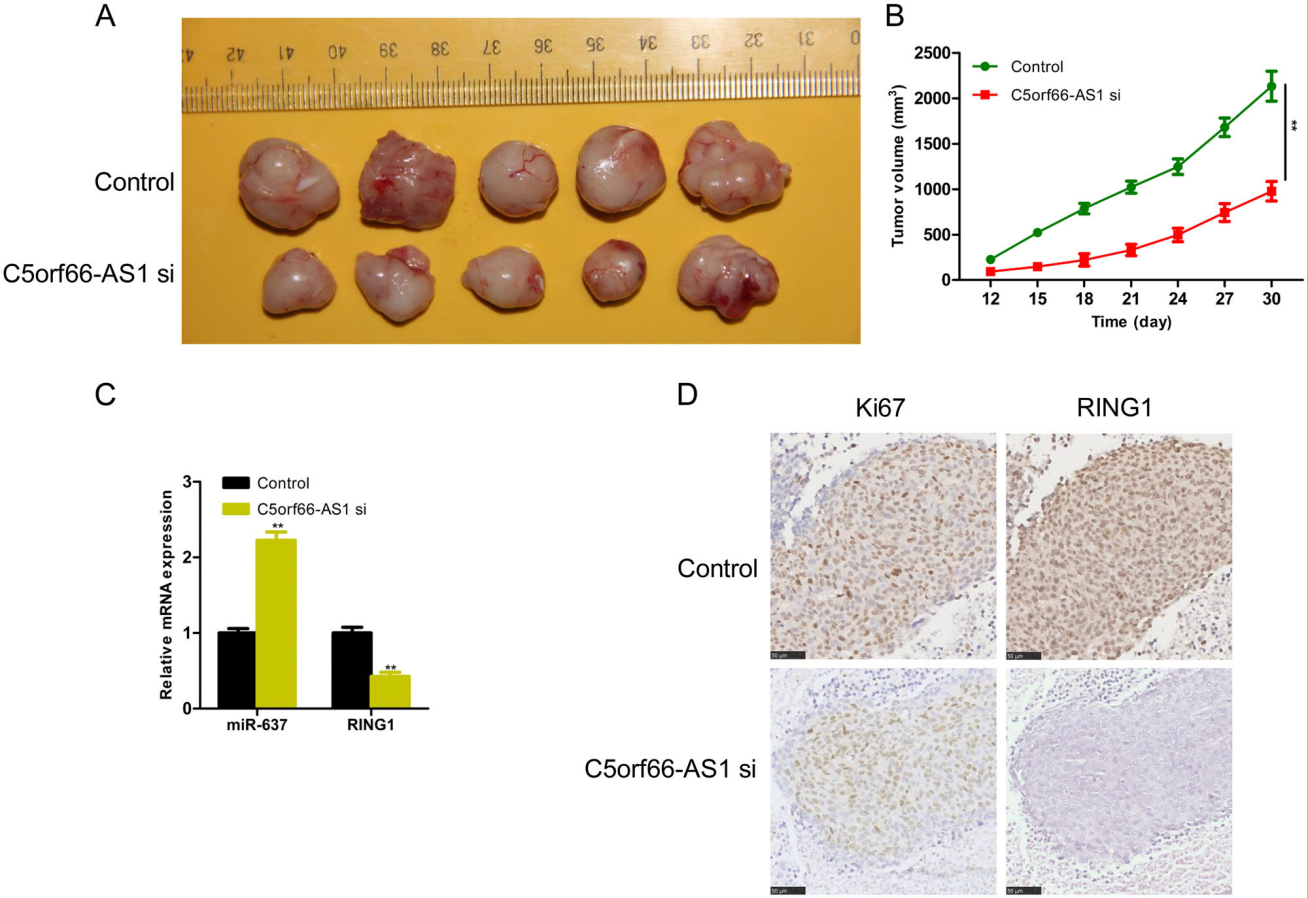
Downregulation of C5orf66-AS1 can inhibit the tumour growth in vivo. **a** Tumours collected for weighting and analyses of gene expression. **b** Growth curves of control and C5orf66-AS1 siRNA. **c** The expression of miR-637 was significantly increased in the C5orf66-AS1-siRNA group, while the expression of RING1 at the mRNA level was significantly decreased in the C5orf66-AS1-siRNA group, as measured via qRT-PCR. **d** Ki67 and RING1 protein levels in tumour tissues from C5orf66-AS1 siRNA or control SiHa cells were determined via immunohistochemistry. ^*^P < 0.05, ^**^P < 0.01, ^***^P < 0.001

To further study the important biological function of C5orf66-AS1 in the occurrence and development of cervical cancer, the subcutaneous tumour-bearing model of cervical cancer was established in nude mice, and the effect of C5orf66-AS1 on the proliferation of cervical cancer cells in animals was further verified. SiHa cells with a stable transfection of C5orf66-AS1 siRNA or control were injected into the back of nude mice in both groups. The growth of subcutaneous tumours in nude mice was observed every 3 days from day 10 after injection, the long and short diameters of the tumours were determined using a Vernier calliper, and the tumour volume was calculated accordingly. Figures 7a, b show that the subcutaneous tumour volume in the C5orf66-AS1 siRNA group began to be significantly smaller compared with the control group from the 15th day after injection. In addition, were also measured the expressions of miR-637 and RING1 in the subcutaneous tumours of both groups. The expression of miR-637 was significantly increased, while that of RING1 was significantly decreased in the C5orf66-AS1-siRNA group (Fig. 7c). Moreover, the immunohistochemistry assay showed that the tumours from the C5orf66-AS1-siRNA group exhibited reduced Ki-67 and RING1 staining compared with the control group (Fig. 7d).

## Discussion

The incidence rate of cervical cancer, one of the most common malignant tumours in females, is second only to that of breast cancer^1^. At present, the research on cervical cancer has developed from the gene level to the protein level, from the cell level to the molecular level, and from in vitro research to in vivo research^11^. The regulatory mechanism of various factors in the pathogenesis of cervical cancer is the greatest challenge in research on this disease. A breakthrough in early diagnosis, prevention and treatment of disease is only likely to be made by starting from these key links. At present, the gene chip technique has been gradually improved with the continuous progress in biotechnology. The gene chip technique can be used to screen cervical cancer-related genes, explore the pathogenesis and study the genetic changes of cervical cancer, providing an important source for the study on the pathogenesis of cervical cancer. Recent studies have aimed to achieve a complete understanding of small non-coding miRNAs in mammalian cells, and satisfactory results have been achieved. However, studies on lncRNA have only exposed the tip of various actions, such as participation in chromatin modification, post-transcriptional regulation, transcriptional activation and inhibition of nuclear transport, thereby regulating cell proliferation, differentiation, metabolism and other pathological processes^12^. Several studies have also confirmed that lncRNA has important biological and pathological functions related to the occurrence and development of tumours^13,14^. Studies have demonstrated that the high expression of HOTAIR can be used as a predictor for poor prognosis of cervical cancer. HOTAIR is highly expressed in cervical cancer tissues and cells, which is correlated with lymph node metastasis, survival rate and postoperative recurrence. At the same time, suppression of lncRNA HOTAIR can reduce the proliferation, migration and invasion of cervical cancer cells and decrease the expressions of some metastasis-associated proteins (VEGF and MMP-9) and EMT-related proteins. LncRNA TPT1-AS1 promotes the proliferation and metastasis of cervical cancer through adsorbing miR-324^15^. LncRNA NCK1-AS1 can promote cell proliferation and induce cell-cycle progression via competitive binding to miR-6857. In this study, we screened 65 lncRNAs that were correlated with cervical cancer using the TCGA database. Next, lncRNA C5orf66 with the most significant differential expression was selected as the object of study via qRT-PCR. Compared with HPV-negative C-33A cells, lncRNA C5orf66-AS1 was highly expressed in HPV-16-positive SiHa cells and HPV-l8-positive C-4 I cells. Therefore, SiHa cells and C-4 I cells were selected for the subsequent study. The in vitro and in vivo experiments demonstrated that C5orf66-AS1 could promote the proliferation of cervical cancer cells. In addition, the nuclear-cytoplasmic fractionation showed that C5orf66-AS1 was mainly distributed in the cytoplasm. Therefore, C5orf66-AS1 might regulate the function of cervical cancer cells through a ceRNA mechanism.

As an endogenous RNA, ceRNA competes with other RNAs for miRNA through miRNA response elements (MREs), affecting the regulation of target genes by miRNA. MiRNA is one of the most studied lncRNAs, and it is closely related to cell proliferation, apoptosis, tumour development and metastasis. At present, there has been increasing research on the correlation between miRNA and cervical cancer, and studies have confirmed that miRNA is closely related to the incidence of cervical cancer. Using bioinformatics and immunofluorescence reporter assay, we confirmed that C5orf66-AS1 and miR-637 could fully bind to each other. MiR-637 was expressed in low levels in cervical cancer tissues and cells compared with the normal tissues and cells, respectively, and it was found to play a role as a tumour suppressor gene. An increasing number of studies have focused on the role of miR-637 in tumours. Zhang et al. have found that miR-637 can inhibit the proliferation of hepatocellular carcinoma (HCC) cells through inhibiting the activation of STAT3^16^. In glioma, low expression of miR-637 is a marker of poor prognosis, and upregulation of miR-637 can significantly inhibit the proliferation, migration and invasion of glioma cells in vitro and in vivo. In pancreatic ductal adenocarcinoma, the over-expression of miR-637 can obviously suppress cell proliferation and induce apoptosis^17^. In addition, miR-637 can inhibit the proliferation of cholangiocarcinoma cells^18^. The above-mentioned findings are consistent with the functions of miR-637 in cervical cancer determined in the present study.

RING1, RNF2 and BMI1 are important components of PRC1. RING1 is over-expressed in various tumours and plays a role as an oncogene in tumours. Xiong et al. have found that downregulation of RING1 can inhibit the proliferation of HCC cells. Shen et al. have revealed that HCC patients with a high expression of RING1 have a poor prognosis, while depletion of RING1 can inhibit the proliferation of wild-type p53 cells via cell-cycle arrest and induce apoptosis^19^. Using bioinformatics and immunofluorescence reporter assay, we confirmed that RING1 was a direct target gene of miR-637. It was highly expressed in cervical cancer tissues and cells, and could promote the proliferation of cervical cancer cells and completely reverse the changes in cell proliferation induced by miR-637.

Collectively, lncRNA C5orf66-AS1, as a ceRNA, regulated the effect of RING1 on the proliferation, apoptosis and cell cycle of cervical cancer through adsorbing miR-637. Our findings provided a new theoretical and experimental basis for investigating the pathogenesis and exploring effective therapeutic targets for cervical cancer.

## Materials and methods

### Tissue specimens

A total of 20 cases of specimens, including paired cervical cancer tissues and para-carcinoma normal tissues, were collected from cervical cancer patients who underwent operation in the Third Affiliated Hospital of Soochow University from 2016 to 2017. The patients were pathologically diagnosed with cervical cancer. The specimens were immediately placed in liquid nitrogen after collection and transferred into a refrigerator at −80 °C. All experiments in this study were approved by the Ethics Committee of the Third Affiliated Hospital of Soochow University. The patients and their families were informed of specimen collection, and they signed the informed consent.

### Cell culture

The normal cervical epithelial cell line End1/E6E7 and the cervical cancer cell lines SiHa, C-4 I, HeLa and C-33A were purchased from the American Type Culture Collection (ATCC, Manassas, VA, USA) and routinely maintained in DMEM supplemented with 1% streptomycin double-antibody and 10% foetal bovine serum at 37 °C in an incubator with 5% CO_2_, followed by a passage once every 2–3 days until cells reached the logarithmic growth phase.

### Construction of transfection plasmid

Small-interfering RNA (siRNA) over-expressing the C5orf66-AS1 and negative control plasmids were purchased from Sigma-Aldrich (Darmstadt, Germany). MiR-637 mimics, miR-637 inhibitors and negative controls were purchased from RiboBio (Guangzhou, China). Lipofectamine 2000 was used as the vector in cell transfection according to the manufacturer’s instructions. At 24 h after transfection, the transfection efficiency was assessed under a fluorescence microscope to determine whether subsequent experiments could be performed. The cells were collected at appropriate time points for further experiments based on the experimental requirements.

### qRT-PCR

Total RNA was extracted from tissues and cells using the TRIzol reagent (Invitrogen, USA). Purified RNA was reversely transcribed into cDNA using a PrimerScript RT Reagent kit (TaKaRa, Japan), and miRNA from total RNA was reversely transcribed using the Prime-Script miRNA cDNA Synthesis Kit (TaKaRa). The amplification was performed on a 7900 FAST real-time PCR instrument using the SYBR Green dye. GAPDH was used as an internal reference for mRNA, while U6 was used as an internal reference for miRNA. The expression levels of mRNAs and miRNAs were calculated using the comparative CT(2^−ΔΔCt^) method. Information regarding the primers is shown in Supplementary Table 1.

### Western blotting analysis

The total protein was extracted from cells and tissues, and the protein concentration was detected using the BCA method. Briefly, 20 μg of protein was subjected to sodium dodecyl sulphate-polyacrylamide gel electrophoresis, then transferred onto a PVDF membrane, blocked with TBST containing 10% skim milk powder at room temperature for 2 h and incubated with the primary antibody at 4 °C overnight. After the membrane was washed with TBST thrice for 10 min each time, the blots were incubated with the corresponding horseradish peroxidase-labelled secondary antibody for 1 h and then washed thrice with TBST again for 10 min each time. Subsequently, the blots were incubated with freshly prepared chemiluminescence solution for 2 min, followed by exposure and image development. GAPDH was used as the loading control.

### Nuclear-cytoplasmic fractionation

The nucleus and cytoplasm of SiHa and C-4 I cells were separated using Nuclear and Cytoplasmic Extraction Reagents (Thermo Scientific, USA), and RNA was extracted for qRT-PCR. GAPDH and U6 served as markers of the cytoplasm and nucleus, respectively.

### Luciferase reporter gene

The binding sites of C5orf66-AS1 and miR-637 were predicted using the database, and the wild-type C5orf66-AS1-WT containing the binding site and C5orf66-AS1-MUT luciferase plasmid containing the binding-site mutation were constructed. The luciferase plasmids C5orf66-AS1-WT and C5orf66-AS1-MUT were co-transfected with miR-637 mimics into SiHa cells. After 48 h, the luciferase activity was detected using the dual-luciferase reporter system according to the instructions.

The binding sites of miR-637 and RING1 3′UTR were predicted through the database, and the wild-type RING1 3′UTR-WT containing the binding site and the RING1 3′UTR-MUT luciferase plasmid containing the binding-site mutation were constructed. The plasmid RING1 3′UTR-WT and RING1 3′UTR-MUT were co-transfected with miR-637 mimics into SiHa cells. After 48 h, the luciferase activity was detected using the dual-luciferase reporter system according to the manufacturer’s instructions.

### RNA immunoprecipitation (RIP) assay

RIP assay was conducted via a Magna RIP RNA-Binding Protein Immunoprecipitation Kit (Millipore, Bedford, MA, USA). Briefly, cultured cells were collected and resuspended in RIP lysis buffer (Solarbio), and the cell extracts were incubated with RIP buffer containing magnetic beads conjugated with human anti-AGO2 antibody (Millipore) or normal mouse IgG (Merck Millipore) overnight. Subsequently, the magnetic beads were incubated with proteinase K after washing three times. Total RNA was subsequently isolated from the extracts using the TRIzol reagent. Lastly, the relative enrichments of C5orf66-AS1 and miR-637 were determined via RT-qPCR analysis.

### Animal experiment

The animal experiment was performed according to the principles and procedures that were stipulated by the Animal Management and Use Committee of Soochow University, and animal-related protocols were approved by the Ethics Committee of the Third Affiliated Hospital of Soochow University. The lncRNA C5orf66-AS1 was depleted in cervical cancer SiHa cells, and the cell lines with stable transfection were screened. Approximately 1 × 10^7^ cells with stable transfection of C5orf66-AS1 siRNA or control plasmid were subcutaneously injected into the back of nude mice. The condition of the mice was observed every 3 days, the long and short diameters of the tumours were recorded, and the tumour volume was calculated accordingly (length × width^2^/2). The nude mice were executed at 30 days after tumour formation, and the specimens were retrieved to measure the tumour volume. The expression levels of C5orf66-AS1, miRNA-637 and RING1 were detected via qRT-PCR.

### Statistical analysis

The SPSS 21.0 (IBM, USA) statistical software was used for statistical analysis in this study. Measurement results were expressed as the mean ± standard deviation (SD). The *t*-test was used for the comparison of continuous variables between two groups, and ANOVA was used for the comparison of continuous variables among groups. Survival data were analyzed using the Kaplan-Meier method and Cox regression. *P* < 0.05 was considered statistically significant.

## Electronic supplementary material

Supplementary table 1
