# Supplementary material for: Long non-coding RNA C5orf66-AS1 promotes cell proliferation in cervical cancer by targeting miR-637/RING1 axis
Source: Cell Death Dis. 2018 Dec 5;9(12):1175. doi: 10.1038/s41419-018-1228-z (PMC6281646; doi:10.1038/s41419-018-1228-z)
Supplement: Supplementary file 1 — Supplementary table 1 [file 41419_2018_1228_MOESM1_ESM.docx]

| Gene | Sequence of the primers |
| --- | --- |
| C5orf66-AS1-Forward | 5'-CGGGATCAACCCTCTGCTTT-3' |
| C5orf66-AS1-Reverse | 5'-TTCTTGAGAAGCGACTGCGT-3' |
| RING1-Forward | 5'-AGAATGCCAGCAAAACGTGG-3' |
| RING1-Reverse | 5'-AGATAGGGCACATGAGTTCTGA-3' |
| SLC8A2-Forward | 5'-TCGAGGTCATCACGTCAAAAG-3' |
| SLC8A2-Reverse | 5'-AGGTTGGACACCGTCTCATTC-3' |
| SPRED3-Forward | 5'-CCAGGGGCACTACGTCATC-3' |
| SPRED3-Reverse | 5'-AACGTCAGTCCAAACTTGCAG-3' |
| TSPAN11-Forward | 5'-CATCTTTGCGGGCGTACTTG-3' |
| TSPAN11-Reverse | 5'-CAGGCAGAAATACGTGGAGAG-3' |
| MNT-Forward | 5'-CCCCACTGACTGTCATCCCTA-3' |
| MNT-Reverse | 5'-GGCAGGCTCCTTAATGCTGAG-3' |
| PVRL1-Forward | 5'-CTCGGCTTGACCGCATTCTT-3' |
| PVRL1-Reverse | 5'-GCAGTGCAGAACCACGTCT-3' |
| GAPDH-Forward | 5'-CTCTCTGCTCTCCTGTTCGAC-3' |
| GAPDH-Reverse | 5'-TGAGCGATGTGGCTCGGCT-3' |

Supplementary table 1 Primer sequences used for qPCR assays
